# Poor Oral Health and Risk of Respiratory Tract Cancer: A Prospective Cohort Study from the UK Biobank

**DOI:** 10.3390/cancers17183028

**Published:** 2025-09-16

**Authors:** Danting Yang, Hyung-Suk Yoon, Tara Hashemian, Young-Rock Hong, Shama D. Karanth, Sai Zhang, Heba El-Ahmad, Shannon M. Wallet, Qiuyin Cai, Xiao-Ou Shu, Ji-Hyun Lee, Jae Jeong Yang

**Affiliations:** 1 Department of Epidemiology, College of Public Health and Health Professions, University of Florida, Gainesville, FL 32611, USA; dantingyang@ufl.edu (D.Y.); sai.zhang@ufl.edu (S.Z.); 2 University of Florida Health Cancer Center, Gainesville, FL 32610, USA; yoon.h@ufl.edu (H.-S.Y.); thashemian@ufl.edu (T.H.); shama.karanth@ufl.edu (S.D.K.); jihyun.lee@ufl.edu (J.-H.L.); 3 Department of Surgery, College of Medicine, University of Florida, Gainesville, FL 32610, USA; swallet@dental.ufl.edu; 4 Department of Family and Preventive Medicine, Emory University, Atlanta, GA 30322, USA; youngrock.hong@emory.edu; 5 Department of Oral Biology, College of Dentistry, University of Florida, Gainesville, FL 32610, USA; hel-ahmad@dental.ufl.edu; 6 Division of Epidemiology, Department of Medicine, Vanderbilt University Medical Center, Nashville, TN 37232, USA; qiuyin.cai@vumc.org (Q.C.); xiao-ou.shu@vumc.org (X.-O.S.); 7 Vanderbilt-Ingram Cancer Center, Vanderbilt University Medical Center, Nashville, TN 37232, USA; 8 Department of Biostatistics, College of Public Health and Health Professions, University of Florida, Gainesville, FL 32611, USA

**Keywords:** oral health, respiratory tract cancer, bronchus and lung cancer, laryngeal cancer, epidemiology, UK Biobank

## Abstract

Background/Objectives: The individual and combined effects of diverse dental problems on site-specific respiratory tract cancer remain insufficiently addressed. This study investigated the associations between six oral conditions—dentures, loose teeth, painful gums, bleeding gums, toothache, and mouth ulcers—and the risk of developing cancers of the lung, bronchus, larynx, and trachea, both individually and collectively. Methods: This prospective cohort study, utilizing baseline data from the UK Biobank, analyzed 438,762 participants aged 40–69 years. Multivariable Cox proportional hazards models were employed to estimate hazard ratios (HRs) and 95% CIs for incident respiratory tract cancer associated with individual oral conditions and their cumulative impact. Effect modification by sociodemographics, lifestyle, and underlying medical conditions was further assessed. Results: During the follow-up (median = 11.0 years), 3568 participants developed respiratory tract cancer. After adjusting for confounders, including smoking history, individuals with any oral issues exhibited a 35–76% higher risk of respiratory tract cancer (HR [95% CI]: 1.35 [1.25–1.46] for the total risk, 1.34 [1.24–1.45] for bronchus/lung cancer, and 1.76 [1.23–2.52] for laryngeal cancer). Dentures (1.48 [1.36–1.60]), loose teeth (1.36 [1.20–1.54]), and painful gums (1.25 [1.04–1.50]) had a strong association with respiratory tract cancer. The overall risk escalated progressively with the number of concurrent problems (HR [95% CI] of 1.32 [1.22–1.43] for one, 1.42 [1.25–1.62] for two, 1.57 [1.23–1.98] for three, and 1.71 [1.16–2.50] for ≥four). Associations were pronounced among smokers (*p*-interaction = 0.002). Conclusions: Poor oral health is linked to a heightened risk of respiratory tract cancer, especially when coupled with smoking, underscoring the importance of oral health promotion in cancer prevention strategies.

## Simple Summary

Poor oral health has been implicated in the risk of various cancers; however, the contribution of specific dental conditions and their cumulative burden on the risk of site-specific respiratory tract cancer—lung, bronchus, larynx, and trachea—remains underexplored. In this population-based cohort study, we analyzed 438,762 participants aged 40–69 years from the UK Biobank to examine the association of six oral conditions, including dentures, loose teeth, painful gums, bleeding gums, toothache, and mouth ulcers, with the risk of developing respiratory tract cancer. Individuals with any oral issues demonstrated a 35–76% higher risk of respiratory tract cancer compared to those without such issues. The risk of respiratory tract cancer escalated progressively with the number of concurrent oral problems, showing effect modification by smoking history. These findings highlight that enhancing dental hygiene practices, along with smoking cessation, can serve as effective public health strategies for the prevention of respiratory tract cancers.

## 1. Introduction

Oral health may play a crucial role in predisposing individuals to systemic inflammatory and immune responses that contribute to carcinogenesis [1,2]. Chronic oral diseases, such as periodontitis, tooth loss, gingival bleeding, and painful gums, constitute indicators of localized oral inflammation but may reflect underlying systemic dysregulation, potentially creating a tumor-promoting environment [1,2,3]. Emerging evidence indicates that oral pathogens—for instance, *P. gingivalis* and *F. nucleatum*—shape the tumor microenvironment by modulating inflammatory signaling pathways, inhibiting immune responses, disrupting tumor suppressor pathways, and producing carcinogenic substances (e.g., nitrosamines and acetaldehyde) [1,2,4,5]. Although there are some discrepancies, epidemiological studies to date have found an association between poor oral health and increased risk of various cancer types, including lung, gastrointestinal system, pancreatic, prostate, and oral cancers [6,7,8,9,10,11,12,13,14,15,16,17,18].

The anatomical and functional connection between the oral cavity and respiratory tract suggests that poor oral health, characterized by oral inflammation, microbial dysbiosis, and periodontal diseases, may directly affect respiratory epithelial cells via inhalation or hematogenous spread, thereby elevating the risk of developing respiratory tract cancer. Some previous studies have reported that tooth loss and/or periodontal diseases are linked to the development of lung cancer, a major type of respiratory tract cancer [11,12,13,14,15]. However, few studies have considered the anatomic specificity of respiratory tract regions—lung/bronchial, laryngeal, and tracheal—nor have they investigated the impact of diverse dental conditions, their severity, and the cumulative burden of such conditions. Furthermore, oral health trajectories can be affected by individuals’ characteristics such as socioeconomic status, lifestyle, and pre-existing health conditions [19,20,21]; however, there is limited understanding of whether these underlying risk factors interfere with the primary association between oral health and respiratory tract cancer risk.

In this population-based cohort study, leveraging the UK Biobank data, we examined the association of six distinct oral conditions, i.e., dentures, loose teeth, painful gums, bleeding gums, toothache, and mouth ulcers, with the risk of developing specific types of respiratory tract cancer. We also investigated the cumulative impacts of these problems using two complementary approaches: (1) a count-based approach that quantified the total number of concurrent oral conditions and (2) a severity-based approach that categorized participants according to their most clinically significant reported condition. In addition, we assessed whether these associations were modified by sociodemographics (i.e., age, sex, education level, and income), lifestyle (i.e., smoking and alcohol use), and underlying medical conditions (i.e., chronic obstructive pulmonary disease (COPD) and obesity).

## 2. Materials and Methods

### 2.1. Data Source and Study Population

This study utilized baseline data from the UK Biobank, a large prospective cohort study that recruited about 502,000 community-dwelling individuals aged 40 to 69 years across the United Kingdom between 2006 and 2010. The detailed study procedures and cohort profile have been documented elsewhere [22,23,24]. After providing written consent, participants in the baseline assessments provided detailed information on their sociodemographics, lifestyle, diet, medical history, and other health-related factors, in addition to undergoing physical measurements and donating biological samples (blood, urine, and saliva) for future research. Study participants have been regularly monitored to confirm their health status through linkage to electronic medical and health-related records. The UK Biobank has obtained approval from the North West Multi-Centre Research Ethics Committee [24].

For the present analysis, we first excluded individuals with a documented history of any cancer prior to enrollment based on self-reported doctor diagnosis or confirmation through data linkage. Individuals missing information, including responses of “prefer not to answer,” on oral health conditions, smoking status, or basic demographic variables (age, sex, and race) were removed. In addition, participants who withdrew their consent during the follow-up period were further excluded. After applying these exclusion criteria, a total of 438,762 participants remained in the final eligible analytic sample (Figure S1). The current study followed the Strengthening the Reporting of Observational Studies in Epidemiology (STROBE) guidelines.

### 2.2. Oral Health Conditions

The primary exposure of interest was the presence of oral health conditions, defined as having any of the following problems: dentures, loose teeth, painful gums, bleeding gums, toothache, and mouth ulcers. We incorporated all self-reported dental problem variables captured in the UK Biobank baseline questionnaire, in which participants indicated that they had any of the listed conditions (multiple choices) or “none of the above.” In addition to assessing individual oral health conditions, we created a composite score to represent the cumulative burden of oral health issues by calculating the number of conditions reported by each participant (0, 1, 2, 3, or ≥4) to evaluate potential dose–response relationships between oral health burden and cancer risk. Given that loose teeth, painful gums, and bleeding gums are validated indicators of periodontal diseases [25,26], we also grouped participants into four groups: those without any conditions, those with mouth ulcers/toothache, those with bleeding gums/painful gums/loose teeth, and those with dentures, according to their most severe reported condition.

### 2.3. Incident Respiratory Tract Cancer Cases

The primary outcome of interest was the incidence of respiratory tract cancer, encompassing cancers of the lung, bronchus, larynx, and trachea, which was ascertained through linkage to national cancer registries [24]. The International Classification of Diseases (ICD) Ninth and Tenth Revision codes were used to identify newly diagnosed respiratory tract cancer and its subtypes (ICD-9: 161, 162.0–162.9 and ICD-10: C32, C33, C34; Table S1). Time-to-event analyses started at the enrollment date and were censored at the earliest occurrence of cancer diagnosis, death, or loss to follow-up. Individuals who did not develop respiratory cancer were considered cancer-free only up to their censoring date, not extending beyond the observation period.

### 2.4. Covariates

Potential confounding factors were identified from previous literature of established or suspected risk factors for respiratory tract cancer and added to the model sequentially. The basic model included age at enrollment (continuous), sex (men and women), self-reported race (White, Black, and Other (Asian and multiracial/ethnic)), and smoking history combined with pack-years (never smoked, former smokers, current smokers with <20 pack-years, current smokers with ≥20 pack-years, and current smokers with missing pack-years). The full model further included educational attainment (those who did not complete high school, those who graduated high school, those who attended vocational school/some college, and those with university degrees and beyond), average total household income after tax (<GBP 18,000, GBP 18,000–GBP 30,999, GBP 31,000–GBP 51,999, GBP 52,000–GBP 100,000, and >GBP 100,000), alcohol consumption (never, 1–3 times per month/occasionally, 1–4 times per week, and daily/almost daily), obesity status defined by body mass index (BMI) (calculated as weight in kilograms divided by height in meters squared (underweight <18.5 kg/m^2^, normal 18.5–24.9 kg/m^2^, overweight 25.0–29.9 kg/m^2^, and obese ≥30 kg/m^2^)), and history of doctor-diagnosed COPD (yes vs. no).

### 2.5. Statistical Analysis

Baseline characteristics of the study population were compared between incident respiratory tract cancer cases and those without respiratory cancer using the Wilcoxon rank sum test for continuous variables and Pearson’s Chi-squared test for categorical variables. The step-down Bonferroni method was employed to correct for multiple comparisons in the analysis of the prevalence of six self-reported oral health conditions. Incidence rates of respiratory tract cancer were calculated by dividing the number of newly diagnosed cases by the corresponding person-years at risk, expressed per 1000 person-years. Cox proportional hazards (PH) models were used to estimate hazard ratios (HRs) and 95% confidence intervals (CIs) for the incidence of respiratory tract cancer linked to oral health conditions, with follow-up time as the time scale. A healthy oral condition (without any problems) was modeled as the reference. The PH assumption was evaluated using Schoenfeld residuals—we confirmed no violation of the assumption. Stratified analyses were performed to assess the effect modification by age group, sex, education level, income, smoking history combined with pack-years, alcohol consumption, obesity, and history of COPD, all initially identified as potential risk factors for respiratory tract cancer. Interaction was evaluated using multiplicative terms of oral conditions and the stratification variables. To enhance model fit in the interaction analysis, backward selection was employed to determine the optimal model by utilizing the lowest Akaike information criterion (AIC) value—this approach allowed us to consider both epidemiological evidence on biological plausibility and statistical criteria. A series of sensitivity analyses were conducted to account for residual confounding due to smoking, such as restricting the analysis to those who never smoked or those who were long-term quitters who had abstained from smoking for more than 10 years, while treating smoking-related deaths [27] as a competing risk. No imputation for the minimal missing data was conducted. All statistical tests were two-sided, and analyses were conducted reproducibly, using SAS version 9.4 (SAS Institute, Cary, NC, USA) and R version 4.4.1.

## 3. Results

Of the 438,762 participants analyzed, 3568 developed respiratory tract cancer during the mean follow-up time of 10.3 years (Table 1). Individuals with respiratory tract cancer, in comparison to their cancer-free counterparts, showed an older mean age (61.5 vs. 56.2 years), lower educational attainment (38.1% vs. 16.6% with less than a high school education), lower income (44.5% vs. 21.9% reporting less than GBP 18,000), a notably higher prevalence of heavy smokers (current smokers with ≥20 pack-years: 33.0% vs. 4.9%), and a more frequent history of COPD (1.3% vs. 0.3%). In terms of respiratory tract cancer subtypes (Table S1), bronchus and lung cancer were the most prevalent, comprising approximately 95% of the total, with laryngeal cancer following behind. Tracheal cancer exhibited an extremely low incidence rate, including less than 1% of cases.

**Figure 1:**
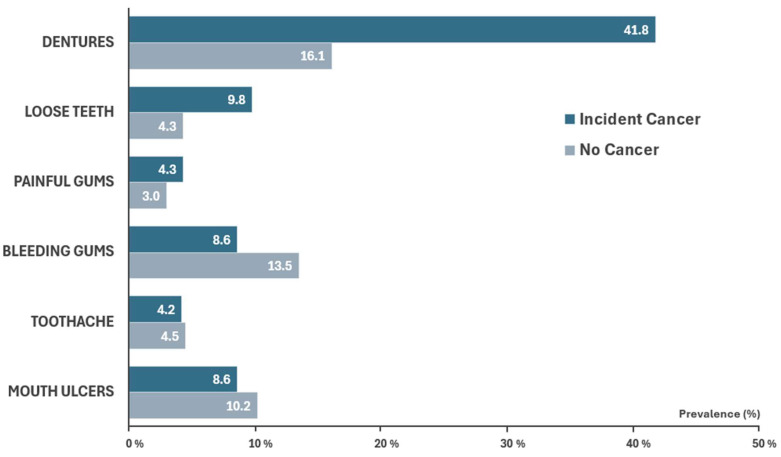
Prevalence (%) of oral health conditions among study participants. The prevalence rates of oral health conditions of participants who developed respiratory tract cancers and those who did not were significantly different after correcting for multiple comparisons (all *p* < 0.01), except for the prevalence of toothache (*p* = 0.36).

The prevalence of oral problems differed significantly between individuals who developed respiratory tract cancer and those who did not (Figure 1 and Table S2). After correcting for multiple comparisons, cancer cases had a significantly higher prevalence of severe conditions, including dentures (41.8% vs. 16.1%), loose teeth (9.8% vs. 4.3%), and painful gums (4.3% vs. 3.0%) with *p* < 0.001 for all. In contrast, bleeding gums (8.6% vs. 13.5%; *p* < 0.001) and mouth ulcers (8.6% vs. 10.2%; *p* = 0.005) were less prevalent among cancer cases, while the prevalence of toothache revealed no significant difference between the groups (4.2% vs. 4.5%; *p* = 0.36). Furthermore, those with respiratory tract cancer were more prone to experiencing one or more oral issues, whereas their cancer-free counterparts were more likely to remain unaffected by such problems (Table S3). Meanwhile, smokers appeared to encounter more severe oral complications than those who had never smoked (Tables S4 and S5)—the prevalence of dentures was substantially higher among current smokers with ≥20 pack-years (34.0%) and former smokers (20.7%), followed by current smokers with <20 pack-years (17.3%), while a prevalence of only 11.8% was observed among those who had never smoked (*p* < 0.001).

After adjusting for all potential confounders, the presence of any oral problems was associated with a 34% to 76% increased risk of developing respiratory tract cancer (Table 2): total respiratory tract (HR = 1.35, 95% CI: 1.25–1.46), bronchus and lung (HR = 1.34, 95% CI: 1.24–1.45), and larynx (HR = 1.76, 95% CI: 1.23–2.52). Regarding individual oral health conditions, dentures were associated with a 1.47- to 1.49-fold increased risk of respiratory tract cancer (HR = 1.48, 95% CI: 1.36–1.60 for total; HR = 1.47, 95% CI: 1.36–1.60 for bronchus and lung; and HR = 1.49, 95% CI: 1.02–2.16 for larynx). Loose teeth and painful gums also significantly elevated the risk of developing respiratory tract cancer, including bronchus and lung cancers (HRs ranged from 1.24 to 1.37). However, no associations were found for bleeding gums, mouth ulcers, or toothache. The associations between oral health and tracheal cancer were not assessed separately due to the very limited number of incident cases.

The risk of respiratory tract cancer increased progressively with the cumulative count of simultaneous oral problems (Table 3). Individuals with a single condition exhibited a 32% increased risk (HR = 1.32, 95% CI: 1.22–1.43) compared to those without any conditions; those having two, three, and four or more conditions experienced a 42%, 57%, and 71% heightened risk, respectively (HR [95% CI] = 1.42 [1.25–1.62], 1.57 [1.23–1.98], and 1.71 [1.16–2.50] from the fully-adjusted model). Individuals exhibiting indications of periodontal diseases (loose teeth, painful gums, or bleeding gums) or using dentures had a 14% and 52% increased risk of respiratory tract cancer, respectively, compared to those without any conditions (HR [95% CI] = 1.14 [1.01–1.29] and 1.52 [1.40–1.66]).

For better model fitting, the race variable was excluded from the final interaction model as it made a minimal contribution to model performance (Table S6). In the final model, the association between oral health and respiratory tract cancer remained generally consistent regardless of underlying risk factors (Table 4)—no significant interactions were found regarding age, sex, education, income, alcohol intake, obesity, and history of COPD. However, the primary association was more pronounced among former smokers (HR = 1.50, 95% CI: 1.34–1.68) and current smokers with <20 pack-years (HR = 1.52, 95% CI: 1.13–2.06) than the other groups (*p*-interaction = 0.002), indicating potential effect modification based on smoking history. Sensitivity analyses restricted to those who had never smoked and long-term quitters or treating smoking-related deaths as a competing risk yielded comparable patterns of association to the primary findings, but with some attenuation of risk estimates (Tables S7 and S8).

## 4. Discussion

In this prospective analysis of 438,762 UK Biobank participants, poor oral health was associated with an increased risk of respiratory tract cancer. After comprehensive adjustments for potential confounders—including smoking status and pack-years—participants with any oral health issues exhibited a 35% higher risk of respiratory tract cancer overall, including a notable 76% increased risk of laryngeal cancer. Among the analyzed oral conditions, utilizing dentures emerged as a strong independent risk factor, conferring a 48% increased risk of respiratory tract cancer overall. A clear dose–response relationship was apparent, with each additional concomitant oral condition further strengthening the risk. The association between oral health and respiratory tract cancer persisted across varying sociodemographics, lifestyles, and pre-existing conditions; however, the association revealed effect modification by smoking history, indicating the potential interplay with smoking or the possibility of residual confounding.

Our findings align with previous studies indicating that tooth loss, periodontal disease, and poor dental hygiene increase the risk of lung cancer [11,12,13,14,15]. A dose–response meta-analysis involving 4052 lung cancer cases and 248,126 non-cases reported that the loss of every five teeth was linked to a 9% increase in the risk of lung cancer, and a 15% increased risk specifically among current smokers [11]. The study further indicated that the risk of lung cancer associated with tooth loss was modified by smoking status, in accordance with the current investigation. Another meta-analysis, pooling results from eight cohorts and four case-control studies, found that periodontitis was significantly associated with a 71% heightened risk of developing lung cancer [15]. Although some conflicting associations were noted in certain populations (e.g., those who had never smoked), severe oral problems appear to be a potential risk factor for lung carcinogenesis. In addition to lung cancer, poor oral health has also been associated with laryngeal cancer patients, as demonstrated by the following studies: (1) the Carolina Head and Neck Cancer Study, a population-based case-control study, reported that tooth loss ranging from 6 to 28 and tooth mobility were associated with a 48% to 53% increased risk of laryngeal cancer, while routine dental visits decreased overall mortality among the cancer patients [17,18]; and (2) a pooled analysis of 865 laryngeal cancer patients from the International Head and Neck Cancer Epidemiology Consortium found that the presence of remaining natural teeth was associated with improved overall survival [28]. Taken together, current epidemiological evidence suggests the potential impact of oral health on lung and laryngeal carcinogenesis, and our findings provide a more expanded perspective of the association between specific oral conditions and respiratory tract cancer, both overall and at particular anatomic sites, indicating their individual and collective effects.

The mechanisms connecting poor oral health to respiratory tract cancers are likely multifactorial. Biologically, certain periodontal conditions contribute to persistent bacteremia, chronic low-grade inflammation, and systemic immunological dysregulation [29,30]. Oral pathogens can spread through the bloodstream, introducing bacterial toxins and byproducts that trigger chronic inflammation [30,31]. The continuous release of pro-inflammatory cytokines such as interleukin-6 and tumor necrosis factor-alpha can directly exacerbate pathologies driven by systemic inflammation and facilitate carcinogenesis by disrupting the host immune system, accelerating cell proliferation, inducing DNA damage, and inhibiting apoptosis [32]. Furthermore, oral dysbiosis—an imbalance in the oral microbial community—also plays an important role in cancer development [33,34]. Particularly, the primary periodontal pathogens, *P. gingivalis* and *F. nucleatum*, are pivotal in triggering tumor-promoting signaling pathways and producing carcinogenic metabolites, such as acetaldehyde, classified as a Group I carcinogen, and N-nitroso compounds formed via nitrate reduction [35,36]. All these oral pathogens might be aspirated into the respiratory tract, making bronchial, lung, and laryngeal tissues susceptible, potentially elevating the risk of respiratory tract cancer. In addition, it is important to note that common risk factors, specifically smoking tobacco, alcohol consumption, and certain viral infections such as human papillomavirus (HPV), contribute to both oral health and respiratory tract cancer. These exposures can cause direct mucosal damage and alter the oral microbiome and immune responses, thereby amplifying the consequences of poor oral health [37,38,39]. Smoking boosts the viral load of HPV [40,41], suggesting an additional mechanistic link beyond bacterial pathogenesis that could influence both oral diseases and respiratory tract cancer, particularly in smokers. This may explain the effect modification by smoking observed in previous epidemiological studies—smokers exhibited more profound associations between poor oral health and lung cancer. Similarly, our study also confirmed a significant interaction with smoking history, indicating that even former smokers with poor oral conditions were strongly linked to a higher risk of respiratory tract cancer. Interestingly, our study found a lower prevalence of bleeding gums among smokers, supporting prior studies on the paradoxical masking effect of smoking that suppresses visible signs of gingival inflammation [42,43]. Such masking may delay the diagnosis and treatment of periodontal disease in ever-smokers, hence increasing the risk of respiratory tract cancer, underscoring the urgent need for integrated public health strategies that address both smoking cessation and periodontal disease management. In addition, low socioeconomic status has now been regarded as an upstream determinant shaping individuals’ oral hygiene, access to dental care, unhealthy habits such as smoking and drinking, and delayed cancer detection [19,20,21,44,45,46,47], although no significant interaction with education or income was observed in our study. Further research is necessary to better understand the biosocial dynamics linking oral health to respiratory tract cancer.

This population-based prospective study introduces several novel findings compared to previous research. Epidemiological studies to date have mainly focused on lung cancer [11,12,13,14,15], with limited investigations of laryngeal cancer, predominantly based on case-control or patient studies [17,18,28]; hence, a comprehensive understanding of respiratory tract cancer is lacking. However, we analyzed respiratory tract cancers both collectively and by specific anatomic sites separately and found a heterogeneity in risk estimates of 1.76 for laryngeal cancer versus 1.34 for bronchus and lung cancer. This distinction may indicate potential site-specific pathophysiological mechanisms, possibly reflecting differential exposure gradients and implicated molecular pathways. In terms of the assessment of oral health, moreover, we systematically characterized six distinct conditions (dentures, loose teeth, painful gums, bleeding gums, toothache, and mouth ulcers) rather than relying on tooth loss/retention as is typical in previous research. This granular approach allowed us to identify the individual impact of each condition and establish a clear dose–response association through both count-based and severity-based analyses. These enhancements strengthen the epidemiological evidence supporting the biological plausibility of the oral–respiratory oncogenic axis.

Our study has several methodological strengths based on a well-characterized cohort of the 438,762 participants analyzed. The extensive survey data and validated long-term follow-up enabled a thorough investigation of diverse oral conditions and various cancer subtypes, both individually and collectively. The prospective design reduces recall bias and strengthens the temporal relationship between oral health conditions and subsequent cancer risk, accounting for a broad spectrum of potential confounders and effect modifiers. However, our study also has limitations that warrant consideration. First, oral health conditions were assessed solely through self-report at baseline, which may introduce non-differential misclassification. Despite the validity and efficacy of self-reported measures for periodontal conditions and dentures in epidemiological studies [25,26,48,49], the lack of clinical validation within the UK Biobank, coupled with the insufficient exploration of the accuracy of other conditions (e.g., toothache and mouth ulcers), might lead to measurement error and misclassification bias, potentially attenuating observed associations. Moreover, the lack of repeated measures on the oral health trajectory restricted our ability to account for temporal changes in oral conditions, including progression, recovery, or interventions, over the follow-up period. Second, residual confounding due to imperfectly measured (cumulative smoking exposure) or unmeasured factors (e.g., oral microbiome composition or HPV infection) cannot be completely ruled out, even when adjusting for a range of covariates. The residual effects of smoking are particularly plausible in light of the inadequate capture of differences in smoking behaviors, such as inhalation depth and type of tobacco products. Our analysis also lacked data on access to dental care and medication use, both of which affect oral conditions. Third, separate analyses of tracheal cancer could not be performed due to its extremely low incidence. When combining tracheal cancer with lung cancer as a standard epidemiological practice, due to their anatomical proximity and analogous etiological pathways, the risk estimates were identical to those observed for lung cancer alone (Table S9). Finally, those belonging to racial and ethnic variations other than White could not be considered as the race variable was removed from the final parsimonious models. Given that more than 94% of UK Biobank participants are of European ancestry, who are known to have higher levels of health awareness and income than the general population [50], the generalizability of our findings may be restricted. Future research should prioritize the incorporation of individuals from racially, ethnically, and socioeconomically diverse backgrounds.

## 5. Conclusions

Our prospective investigation in a large population-based cohort demonstrates that poor oral health is linked to an increased risk of respiratory tract cancer independent of potential underlying risk factors. Given the modifiable and evolving nature of oral pathologies, improving dental hygiene practices, implementing lifestyle modifications, and promoting smoking cessation could serve as proactive cancer prevention strategies for at-risk populations. Further investigations integrating clinical oral assessments, longitudinal data collection, and microbial profiling are needed to enhance our understanding of the biological pathways linking oral health to respiratory tract carcinogenesis, which will pave the way for the development of oral health interventions aimed at cancer prevention.

## Figures and Tables

**Table 1:** Baseline characteristics of study participants.

		Respiratory Tract Cancer		
Characteristics	Total *N* = 438,762	Incident Cancer *N* = 3568	No Cancer *N* = 435,194	*p* ^a^
Follow-up period, years, mean (sd)	10.3 (2.7)	6.6 (3.4)	10.4 (2.6)	<0.001
Age, years, mean (sd)	56.2 (8.1)	61.5 (5.9)	56.2 (8.1)	<0.001
Sex, n (%)				
Women	233,176 (53.1)	1645 (46.1)	231,531 (53.2)	<0.001
Men	205,586 (46.9)	1923 (53.9)	203,663 (46.8)	
Race, ^b^ n (%)				<0.001
White	414,145 (94.4)	3464 (97.1)	410,681 (94.4)	
Black	7371 (1.7)	30 (0.8)	7341 (1.7)	
Other	17,246 (3.9)	74 (2.1)	17,172 (3.9)	
Education, ^c^ n (%)				<0.001
Did not complete high school	72,869 (16.8)	1342 (38.1)	71,527 (16.6)	
Completed high school	73,451 (16.9)	529 (15.0)	72,922 (16.9)	
Vocational school or some college	144,298 (33.2)	1061 (30.1)	143,237 (33.2)	
University and beyond	143,845 (33.1)	589 (16.7)	143,256 (33.2)	
Missing or unknown	4299	47	4252	
Income, n (%)				<0.001
Less than GBP 18,000	83,148 (22.0)	1290 (44.5)	81,858 (21.9)	
GBP 18,000 to GBP 30,999	94,819 (25.1)	832 (28.7)	93,987 (25.1)	
GBP 31,000 to GBP 51,999	99,582 (26.4)	469 (16.2)	99,113 (26.5)	
GBP 52,000 to GBP 100,000	78,841 (20.9)	241 (8.3)	78,600 (21.0)	
Greater than GBP 100,000	20,973 (5.6)	67 (2.3)	20,906 (5.6)	
Missing or unknown	61,399	669	60,730	
Smoking status with pack-years, n (%)				<0.001
Never smoked	242,665 (55.3)	486 (13.6)	242,179 (55.6)	
Former smokers	149,898 (34.2)	1580 (44.3)	148,318 (34.1)	
Current smokers with <20 pack-years	14,391 (3.3)	221 (6.2)	14,170 (3.3)	
Current smokers with ≥20 pack-years	22,321 (5.1)	1177 (33.0)	21,144 (4.9)	
Current smokers missing pack-years	9487 (2.2)	104 (2.9)	9383 (2.2)	
Alcohol consumption, n (%)				<0.001
Never	34,827 (7.9)	370 (10.4)	34,457 (7.9)	
1–3 times per month or occasionally	98,749 (22.5)	798 (22.4)	97,951 (22.5)	
1–4 times per week	215,720 (49.2)	1476 (41.5)	214,244 (49.3)	
Daily or almost daily	89,144 (20.3)	916 (25.7)	88,228 (20.3)	
Missing or unknown	322	8	314	
Obesity status, ^d^ n (%)				<0.001
Underweight	2188 (0.5)	38 (1.1)	2150 (0.5)	
Normal	141,510 (32.4)	1099 (31.1)	140,411 (32.4)	
Overweight	186,181 (42.7)	1488 (42.1)	184,693 (42.7)	
Obese	106,629 (24.4)	911 (25.8)	105,718 (24.4)	
Missing or unknown	2254	32	2223	
History of COPD, n (%)	1518 (0.3)	46 (1.3)	1472 (0.3)	<0.001

Abbreviations: COPD, chronic obstructive pulmonary disease; GBP, Great British Pound; N, number; sd, standard deviation. ^a^: Based on the Wilcoxon rank sum test for continuous variables and Pearson’s Chi-squared test for categorical variables. ^b^: White included British, Irish, White, and any other white background; Black included African, Black, Black British, or Caribbean; and the remaining races and/or ethnicities were classified as Other. ^c^: “Completed high school” included those with CSEs or equivalent and O levels/GCSEs or equivalent; “vocational school or some college” indicated those with NCQ/HND/HNC or equivalent, other professional qualifications (e.g., nursing or teaching), and A levels/AS levels or equivalent; and those with a college or university degree were classified as “university and beyond.” ^d^: Based on body mass index, calculated as weight in kilograms divided by height in meters squared: <18.5 (underweight), ≥18.5 to <25 (normal), ≥25 to <30 (overweight), and ≥30 (obese).

**Table 2:** Association between oral health conditions and respiratory tract cancer and its subtypes.

	Cases, *n*	Person-Years	Incidence Rate ^a^	Hazard Ratio (95% CI) ^b^	Hazard Ratio (95% CI) ^c^
**Respiratory Tract Cancer**					
Any Oral Health Condition	2098	1,783,863	1.18	1.50 (1.40–1.60)	1.35 (1.25–1.46)
Dentures	1492	717,299	2.08	1.63 (1.52–1.75)	1.48 (1.36–1.60)
Loose teeth	348	193,176	1.80	1.42 (1.27–1.59)	1.36 (1.20–1.54)
Painful gums	155	137,030	1.13	1.31 (1.11–1.54)	1.25 (1.04–1.50)
Bleeding gums	306	619,621	0.49	0.97 (0.86–1.09)	0.98 (0.86–1.12)
Toothache	150	205,971	0.73	1.01 (0.86–1.19)	0.95 (0.79–1.15)
Mouth ulcers	308	461,821	0.67	1.08 (0.96–1.22)	1.03 (0.90–1.17)
**Bronchus and Lung Cancer**					
Any Oral Health Condition	2000	1,783,247	1.12	1.49 (1.39–1.59)	1.34 (1.24–1.45)
Dentures	1428	716,878	1.99	1.63 (1.52–1.75)	1.47 (1.36–1.60)
Loose teeth	337	193,109	1.75	1.45 (1.29–1.62)	1.37 (1.21–1.56)
Painful gums	148	137,007	1.08	1.30 (1.10–1.54)	1.24 (1.03–1.49)
Bleeding gums	292	619,546	0.47	0.96 (0.85–1.09)	0.98 (0.85–1.12)
Toothache	140	205,907	0.68	1.00 (0.84–1.18)	0.93 (0.76–1.13)
Mouth ulcers	295	461,750	0.64	1.08 (0.96–1.22)	1.02 (0.89–1.17)
**Laryngeal Cancer**					
Any Oral Health Condition	96	1,770,596	0.05	1.76 (1.28–2.43)	1.76 (1.23–2.52)
Dentures	62	707,930	0.09	1.64 (1.17–2.29)	1.49 (1.02–2.16)
Loose teeth	11	190,968	0.06	0.94 (0.51–1.74)	1.10 (0.59–2.06)
Painful gums	7	136,049	0.05	1.53 (0.71–3.26)	1.49 (0.66–3.40)
Bleeding gums	14	617,830	0.02	1.09 (0.62–1.90)	1.13 (0.62–2.07)
Toothache	10	204,992	0.05	1.32 (0.69–2.51)	1.42 (0.72–2.80)
Mouth ulcers	13	459,898	0.03	1.16 (0.65–2.05)	1.17 (0.63–2.18)

Tracheal cancer was not reported separately due to the extremely low incidence, with fewer than five cases. ^a^: Computed per 1000 person-years. ^b^: HR from multivariable Cox proportional hazards model, adjusted for age at enrollment, sex, race, and smoking history combined with pack-year. ^c^: HR from multivariable Cox proportional hazards model, adjusted for age at enrollment, sex, race, smoking history combined with pack-years, educational attainment, household income, alcohol consumption, obesity status, and history of chronic obstructive pulmonary disease.

**Table 3:** Association between oral health conditions and respiratory tract cancer.

	Cases, *n*	Person-Years	Incidence Rate ^a^	Hazard Ratio (95% CI) ^b^	Hazard Ratio (95% CI) ^c^
**Number of Existing Conditions**					
None	1470	2,756,088	0.53	1 (reference)	1 (reference)
1	1603	1,365,174	1.17	1.46 (1.36–1.57)	1.32 (1.22–1.43)
2	370	318,934	1.16	1.58 (1.41–1.77)	1.42 (1.25–1.62)
3	90	74,911	1.20	1.71 (1.38–2.12)	1.57 (1.23–1.98)
4+	35	24,844	1.41	1.96 (1.40–2.73)	1.71 (1.16–2.50)
**Severity of Poor Oral Health ^d^**					
None	1470	2,756,088	0.53	1 (reference)	1 (reference)
Mouth ulcers, toothache	180	361,327	0.50	1.11 (0.95–1.29)	1.04 (0.88–1.24)
Bleeding/painful gums, loose teeth	426	705,237	0.60	1.22 (1.10–1.36)	1.14 (1.01–1.29)
Dentures	1492	717,299	2.08	1.71 (1.59–1.84)	1.52 (1.40–1.66)

^a^: Computed per 1000 person-years. ^b^: HRs from multivariable Cox proportional hazards model, adjusted for age at enrollment, sex, race, and smoking history combined with pack-years. ^c^: HRs from multivariable Cox proportional hazards model, adjusted for age at enrollment, sex, race, smoking history combined with pack-years, educational attainment, household income, alcohol consumption, obesity status, and history of chronic obstructive pulmonary disease. ^d^: Patients with multiple conditions were categorized based on their most severe condition reported: none, mouth ulcers/toothache (less severe), bleeding gums/painful gums/loose teeth, and dentures (most severe).

**Table 4:** Association between the presence of oral health conditions and the risk of respiratory tract cancer across subgroups.

	Cases, *n*	Person-Years	Incidence Rate ^a^	Hazard Ratio (95% CI) ^b^	*p*_interaction_ ^c^
Age group, ^d^ years					
<50	171	1,182,777	0.14	1.30 (0.94–1.82)	0.51
50–57	628	1,213,256	0.52	1.39 (1.17–1.65)	
58–62	960	1,010,400	0.95	1.37 (1.19–1.59)	
≥63	1809	1,133,518	1.60	1.38 (1.23–1.54)	
Sex					
Women	1645	2,440,333	0.67	1.38 (1.23–1.55)	0.71
Men	1923	2,099,618	0.92	1.33 (1.20–1.47)	
Education					
Did not complete high school	1342	736,027	1.82	1.46 (1.27–1.67)	0.20
Completed high school	529	760,716	0.70	1.40 (1.15–1.69)	
Vocational school or some college	1061	1,495,759	0.71	1.36 (1.19–1.55)	
University and beyond	589	1,503,512	0.39	1.13 (0.95–1.35)	
Income					
Less than GBP 18,000	1290	840,269	1.54	1.53 (1.36–1.72)	0.14
GBP 18,000 to GBP 30,999	832	971,601	0.86	1.29 (1.12–1.49)	
GBP 31,000 to GBP 51,999	469	1,042,460	0.45	1.11 (0.92–1.33)	
GBP 52,000 to GBP 100,000	241	834,354	0.29	1.24 (0.96–1.61)	
Greater than GBP 100,000	67	221,718	0.30	1.45 (0.88–2.39)	
Smoking Status with Pack-Years					
Never smoked	486	2,539,171	0.19	1.12 (0.91–1.39)	0.002
Former smokers	1580	1,529,466	1.03	1.50 (1.34–1.68)	
Current smokers with <20 pack-years	221	150,382	1.47	1.52 (1.13–2.06)	
Current smokers with ≥20 pack-years	1177	222,344	5.29	1.22 (1.06–1.39)	
Current smokers missing pack-years	104	98,588	1.05	1.18 (0.74–1.86)	
Alcohol Consumption					
Never	370	357,896	1.03	1.24 (0.95–1.60)	0.78
1–3 times per month or occasionally	798	1,024,421	0.78	1.52 (1.29–1.80)	
1–4 times per week	1476	2,242,781	0.66	1.31 (1.16–1.47)	
Daily or almost daily	916	911,572	1.00	1.33 (1.15–1.54)	
Obesity Status					
Underweight	38	22,382	1.70	1.41 (0.60–3.32)	0.99
Normal	1099	1,477,722	0.74	1.32 (1.15–1.51)	
Overweight	1488	1,923,046	0.77	1.34 (1.19–1.51)	
Obese	911	1,094,892	0.83	1.39 (1.20–1.62)	
History of COPD					
Yes	46	15,306	3.01	1.40 (0.75–2.64)	0.83
No	3522	4,524,645	0.78	1.35 (1.25–1.46)	

Abbreviations: COPD, chronic obstructive pulmonary disease; GBP, Great British Pound; N, number. ^a^: Computed per 1000 person-years. ^b^: HR from multivariable Cox PH model, adjusted for age at enrollment, sex, smoking status, educational attainment, household income, alcohol consumption, obesity status, and history of chronic obstructive pulmonary disease. ^c^: Interaction of the presence of any oral health condition and stratification variables. ^d^: Categorized based on the 25th, 50th, and 75th percentiles of the overall patient population.

## Data Availability

UK Biobank data are accessible to researchers following the approval of a formal application via the UK Biobank Access Management System. All other data supporting the findings of this study will be available upon reasonable request to the corresponding author.

## Supplementary Materials

The following supporting information can be downloaded at https://www.mdpi.com/article/10.3390/cancers17183028/s1, Figure S1: Flowchart for Selection of Study Population; Table S1: Definition and Distribution of Outcomes; Table S2: Baseline Prevalence of Oral Health Conditions among Study Participants; Table S3: Number of Oral Health Conditions Stratified by Cancer Status; Table S4: Prevalence of Oral Health Conditions Stratified by Smoking Status; Table S5: Number of Oral Health Conditions Stratified by Smoking Status; Table S6: Multivariable Cox Regression Models for Respiratory Tract Cancer: Adjusted for Different Covariates; Table S7: Sensitivity Analyses: Association Between Oral Health Conditions and Respiratory Tract Cancer; Table S8: Sensitivity Analyses: Association Between Oral Health and Respiratory Tract Cancer; Table S9: Sensitivity Analyses Combining Tracheal Cancer with Lung Cancer.

## Abbreviations

AIC	Akaike information criterion
BMI	body mass index
COPD	chronic obstructive pulmonary disease
Cox PH model	Cox proportional hazards model
HPV	human papillomavirus
HR	hazard ratio
ICD	International Classification of Diseases
PH assumption	proportional hazards assumption
STROBE	Strengthening the Reporting of Observational Studies in Epidemiology
95% CI	95% confidence interval
