# Supplementary material for: Poor Oral Health and Risk of Respiratory Tract Cancer: A Prospective Cohort Study from the UK Biobank
Source: Cancers (Basel). 2025 Sep 16;17(18):3028. doi: 10.3390/cancers17183028 (PMC12468644; doi:10.3390/cancers17183028)
Supplement: Supplementary file 1 [file cancers-17-03028-s001.zip › cancers-3842945-supplementary.pdf]

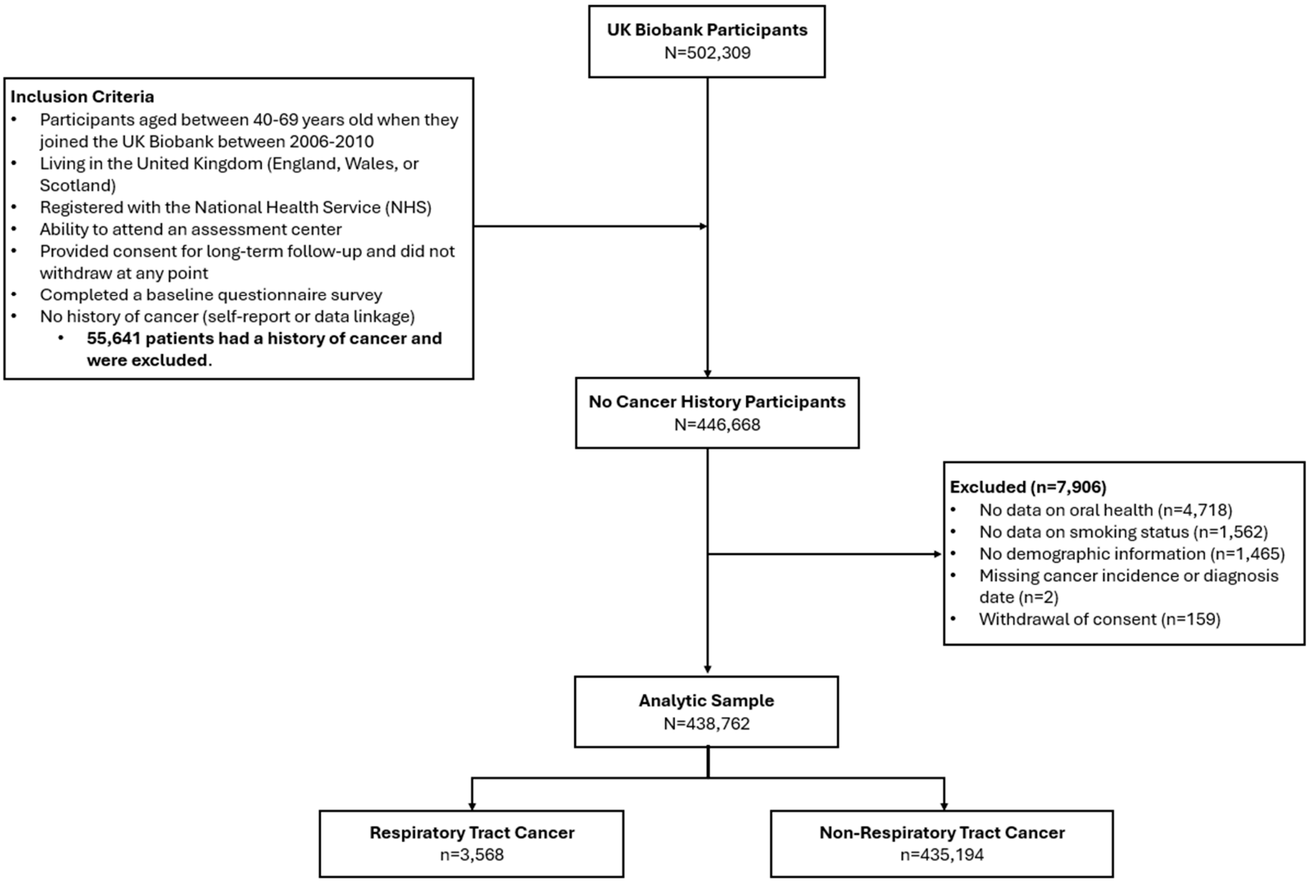

Figure S1. Flowchart for Selection of Study Population

Table S1. Definition and Distribution of Outcomes [a]

	ICD 10	ICD 9	N
**Respiratory Tract Cancer**	C32, C33, C34	161, 162	3,568
**Bronchus and Lung**	C34	162.2, 162.3, 162.4, 162.5, 162.8, 162.9	3,403
**Larynx**	C32	161	161
**Trachea**	C33	162.0	4

a. All are exclusive—no duplicated diagnosis

Table S2. Baseline Prevalence of Oral Health Conditions among Study Participants

Oral Health Conditions	Total Participants, *N*=438,762			
	Respiratory Tract Cancer *n*=3,568	Non-Respiratory Tract Cancer *n*=435,194	*P* [a]	*P* [b]
Dentures	1,492 (41.8%)	69,882 (16.1%)	<0.001	<0.001
Loose teeth	348 (9.8%)	18,621 (4.3%)	<0.001	<0.001
Painful gums	155 (4.3%)	13,147 (3.0%)	<0.001	<0.001
Bleeding gums	306 (8.6%)	58,962 (13.5%)	<0.001	<0.001
Toothache	150 (4.2%)	19,689 (4.5%)	0.36	0.36
Mouth ulcers	308 (8.6%)	44,196 (10.2%)	0.003	0.005
None	1,470 (41.2%)	263,763(60.6%)	<0.001	<0.001

a. Raw *p*-value from Pearson's Chi-squared test
b. Corrected *p*-value using the Step-down Bonferroni method

Table S3. Number of Oral Health Conditions Stratified by Cancer Status

Number of Conditions	Total Participants, *N*=438,762		
	Respiratory Tract Cancer *n*=3,568	Non-Respiratory Tract Cancer *n*=435,194	*P* [a]
0	1,470 (41%)	263,763 (61%)	<0.001
1	1,603 (45%)	131,150 (30%)	
2	370 (10%)	30,659 (7.0%)	
3	90 (2.5%)	7,222 (1.7%)	
4	30 (0.8%)	1,755 (0.4%)	
5	4 (0.1%)	527 (0.1%)	
6	1 (<0.1%)	118 (<0.1%)	

a. Kruskal-Wallis rank sum test

Table S4. Prevalence of Oral Health Conditions Stratified by Smoking Status

Oral Health Conditions	Total Participants, *N*=438,762						
	**Never Smoked *n*=242,665**	**Former Smokers *n*=149,898**	**Current <20 PY *n*=14,391**	**Current ≥20 PY *n*=22,321**	**Current Missing PY *n*=9,487**	***P*** [a]	***P*** [b]
Dentures	28,746 (11.8%)	31,016 (20.7%)	2,495 (17.3%)	7,580 (34.0%)	1,537 (16.2%)	<0.001	<0.001
Loose teeth	6,779 (2.8%)	7,923 (5.3%)	1,129 (7.8%)	2,651 (12%)	487 (5.1%)	<0.001	<0.001
Painful gums	6,575 (2.7%)	4,728 (3.2%)	615 (4.3%)	1,093 (4.9%)	291 (3.1%)	<0.001	<0.001
Bleeding gums	33,437 (13.8%)	21,438 (14.3%)	1,496 (10.4%)	1,711 (7.7%)	1,186 (12.5%)	<0.001	<0.001
Toothache	10,386 (4.3%)	6,473 (4.3%)	957 (6.6%)	1,503 (6.7%)	520 (5.5%)	<0.001	<0.001
Mouth ulcers	25,737 (10.6%)	15,739 (10.5%)	910 (6.3%)	1,318 (5.9%)	800 (8.4%)	<0.001	<0.001
None	155,994 (64.3%)	84,179 (56.2%)	8,777 (61.0%)	10,521 (47.1%)	5,762 (60.7%)	<0.001	<0.001

Abbreviations: PY, pack-years.

a. Raw *p*-value from Pearson's Chi-squared test
b. Corrected *p*-value using the Step-down Bonferroni method

Table S5. Number of Oral Health Conditions Stratified by Smoking Status

Number of Conditions	Total Participants, *N*=438,762					
	**Never Smoked *n*=242,665**	**Former Smokers *n*=149,898**	**Current <20 PY *n*=14,391**	**Current ≥20 PY *n*=22,321**	**Current Missing PY *n*=9,487**	***P*** [a]
0	155,994 (64%)	84,179 (56%)	8,777 (61%)	10,521 (47%)	5,762 (61%)	<0.001
1	67,262 (28%)	49,501 (33%)	4,164 (29%)	8,923 (40%)	2,903 (31%)	
2	15,080 (6.2%)	12,214 (8.1%)	1,059 (7.4%)	2,061 (9.2%)	615 (6.5%)	
3	3,361 (1.4%)	2,964 (2.0%)	279 (1.9%)	547 (2.5%)	161 (1.7%)	
4	723 (0.3%)	762 (0.5%)	83 (0.6%)	188 (0.8%)	29 (0.3%)	
5	207 (<0.1%)	220 (0.1%)	23 (0.2%)	68 (0.3%)	13 (0.1%)	
6	38 (<0.1%)	58 (<0.1%)	6 (<0.1%)	13 (<0.1%)	4 (<0.1%)	

Abbreviation: PY, pack-years.

a. Kruskal-Wallis rank sum test

Table S6. Multivariable Cox Regression Models for Respiratory Tract Cancer: Adjusted for Different Covariates

Variables	Model 1 [a] HR (95% CI)	Model 2 [b] HR (95% CI)	Model 3 [c] HR (95% CI)
Presence of Oral Health Conditions [ref: No]	1.50 (1.40-1.60)	1.35 (1.25-1.46)	1.35 (1.25-1.46)
Age	1.11 (1.10-1.12)	1.10 (1.09-1.10)	1.10 (1.09-1.10)
Sex: Female [ref: Male]	0.92 (0.86-0.98)	0.88 (0.82-0.95)	0.89 (0.82-0.96)
Race: Blacks [ref: Whites]	1.03 (0.72-1.48)	0.98 (0.64-1.51)	—
Race: Others [ref: Whites]	0.87 (0.69-1.10)	0.84 (0.64-1.11)	—
Education: Completed high school [ref: <HS]	—	0.75 (0.67-0.85)	0.75 (0.67-0.85)
Education: Vocational/some college [ref: <HS]	—	0.79 (0.72-0.87)	0.79 (0.71-0.86)
Education: ≥University [ref: <HS]	—	0.61 (0.54-0.69)	0.61 (0.54-0.68)
Income: £18,000 to £30,999 [ref: <£18,000]	—	0.85 (0.77-0.93)	0.85 (0.77-0.93)
Income: £31,000 to £51,999 [ref: <£18,000]	—	0.69 (0.61-0.77)	0.69 (0.62-0.78)
Income: £52,000 to £100,000 [ref: <£18,000]	—	0.64 (0.55-0.74)	0.64 (0.55-0.75)
Income: Greater than £100,000 [ref: <£18,000]	—	0.79 (0.61-1.02)	0.79 (0.61-1.02)
Smoking: Former smokers [ref: Never]	4.26 (3.85-4.72)	4.20 (3.73-4.72)	4.21 (3.74-4.73)
Smoking: Current smokers <20 PY [ref: Never]	10.68 (9.10-12.53)	10.09 (8.44-12.06)	10.08 (8.43-12.05)
Smoking: Current smokers ≥20 PY [ref: Never]	25.77 (23.15-28.69)	22.77 (20.11-25.78)	22.85 (20.18-25.86)
Smoking: Current smokers missing PY [ref: Never]	5.91 (4.78-7.31)	5.36 (4.18-6.86)	5.35 (4.18-6.86)
Alcohol: 1-3 times per month [ref: Never]	—	0.83 (0.72-0.96)	0.83 (0.72-0.96)
Alcohol: 1-4 times per week [ref: Never]	—	0.82 (0.72-0.94)	0.83 (0.72-0.95)
Alcohol: Daily or almost daily [ref: Never]	—	0.91 (0.79-1.06)	0.92 (0.80-1.06)
Obesity Status: Underweight [ref: Normal]	—	1.30 (0.88-1.91)	1.30 (0.88-1.91)
Obesity Status: Overweight [ref: Normal]	—	0.86 (0.79-0.94)	0.86 (0.79-0.94)
Obesity Status: Obese [ref: Normal]	—	0.91 (0.82-1.00)	0.91 (0.82-1.01)
COPD: History of COPD [ref: No]	—	1.63 (1.21-2.20)	1.64 (1.21-2.21)
**AIC Value**	**84788.4**	**66488.5**	**66486.1**

Abbreviations: HR, hazard ratio; 95% CI, 95% confidence interval; ref, reference; HS, high school; PY, pack-years; COPD, chronic obstructive pulmonary disease; AIC, Akaike information criterion.

a. Adjusted for age at enrollment, sex, race, and smoking history combined with pack-years
b. Adjusted for age at enrollment, sex, race, smoking history combined with pack-years, educational attainment, household income, alcohol consumption, obesity status, and history of chronic obstructive pulmonary disease
c. After backwards selection, the race variable was removed and adjusted for age at enrollment, sex, smoking history combined with pack-years, educational attainment, household income, alcohol consumption, obesity status, and history of chronic obstructive pulmonary disease

Table S7. Sensitivity Analyses: Association Between Oral Health Conditions and Respiratory Tract Cancer

	Cases, *n*	Person-years	Incidence Rate [a]	Hazard Ratio (95% CI) [b]	Hazard Ratio (95% CI) [c]
Never Smokers, n=242,665					
Any Oral Health Condition	210	904,568	0.23	1.21 (1.01-1.45)	1.12 (0.91-1.38)
Dentures	108	295,280	0.37	1.35 (1.08-1.69)	1.22 (0.93-1.60)
Loose teeth	18	70,520	0.26	1.13 (0.70-1.81)	1.12 (0.65-1.96)
Painful gums	11	68,882	0.16	0.81 (0.45-1.48)	0.80 (0.40-1.61)
Bleeding gums	57	351,984	0.16	1.00 (0.76-1.32)	1.11 (0.82-1.51)
Toothache	24	108,994	0.22	1.32 (0.87-1.99)	1.15 (0.69-1.90)
Mouth ulcers	55	269,462	0.20	1.15 (0.87-1.52)	1.04 (0.74-1.45)
Long-term Quitters, [d] ***n=72,096***					
Any Oral Health Condition	408	323437	1.26	1.43 (1.23-1.65)	1.30 (1.10-1.54)
Dentures	283	162858	1.74	1.53 (1.31-1.79)	1.39 (1.16-1.66)
Loose teeth	45	37140	1.21	1.18 (0.87-1.60)	1.06 (0.75-1.49)
Painful gums	26	22099	1.18	1.21 (0.82-1.79)	1.14 (0.73-1.78)
Bleeding gums	74	96367	0.77	1.00 (0.79-1.28)	0.97 (0.74-1.28)
Toothache	22	29655	0.74	0.88 (0.57-1.34)	0.94 (0.59-1.48)
Mouth ulcers	68	78045	0.87	0.93 (0.72-1.19)	0.90 (0.68-1.20)
Competing Risk Model, [e] ***n=438,762***					
Any Oral Health Condition	2,098	1,783,863	1.18	1.49 (1.39-1.59)	1.35 (1.25-1.45)
Dentures	1,492	717,299	2.08	1.62 (1.51-1.74)	1.47 (1.35-1.59)
Loose teeth	348	193,176	1.80	1.42 (1.27-1.59)	1.36 (1.20-1.54)
Painful gums	155	137,030	1.13	1.30 (1.11-1.53)	1.25 (1.04-1.50)
Bleeding gums	306	619,621	0.49	0.97 (0.86-1.09)	0.98 (0.86-1.12)
Toothache	150	205,971	0.73	1.01 (0.86-1.19)	0.95 (0.79-1.15)
Mouth ulcers	308	461,821	0.67	1.08 (0.96-1.21)	1.020.89-1.17)

a. Computed per 1,000 person-years
b. HR from multivariable Cox proportional hazards model, adjusted for age at enrollment, sex, race, and smoking history combined with pack-years
c. HR from multivariable Cox proportional hazards model, adjusted for age at enrollment, sex, race, smoking history combined with pack-years, educational attainment, household income, alcohol consumption, obesity status, and history of chronic obstructive pulmonary disease
d. Former smokers who had abstained from smoking for more than 10 years
e. Treating smoking-related deaths as a competing risk; smoking-related mortality encompasses cancers of the lung, lip, pharynx, oral cavity, pharynx, esophagus, stomach, pancreas, larynx, cervix uteri (in women), kidney and renal pelvis, bladder, liver, colon, and rectum, and acute myeloid leukemia, as well as coronary heart disease, rheumatic heart disease, pulmonary heart disease, other forms of heart diseases, cerebrovascular disease, atherosclerosis, aortic aneurysm, additional arterial diseases, pneumonia, influenza, tuberculosis, and chronic obstructive pulmonary disease including emphysema, bronchitis, and chronic airways obstruction

Table S8. Sensitivity Analyses: Association Between Oral Health and Respiratory Tract Cancer

	Cases, *n*	Person-years	Incidence Rate [a]	Hazard Ratio (95% CI) [b]	Hazard Ratio (95% CI) [c]
Never Smokers, n=242,665					
Number of Existing Conditions					
None	276	1634603	0.17	1 (reference)	1 (reference)
1	163	702325	0.23	1.20 (0.98-1.45)	1.08 (0.86-1.36)
2	34	157038	0.22	1.17 (0.81-1.66)	1.19 (0.80-1.78)
3	10	35079	0.29	1.60 (0.85-3.02)	1.65 (0.81-3.35)
4+	3	10126	0.30	- [d]	- [d]
Severity of Poor Oral Health [e]					
None	276	1634603	0.17	1 (reference)	1 (reference)
Mouth Ulcers, Toothache	41	220225	0.19	1.17 (0.84-1.62)	1.00 (0.67-1.47)
Bleeding/Painful gums, Loose teeth	61	389063	0.16	1.02 (0.77-1.35)	1.07 (0.79-1.45)
Dentures	108	295280	0.37	1.38 (1.10-1.73)	1.23 (0.94-1.63)
Long-term Quitters,[f] n=72,096					
Number of Existing Conditions					
None	304	406641	0.75	1 (reference)	1 (reference)
1	321	245163	1.31	1.46 (1.24-1.71)	1.34 (1.12-1.60)
2	70	59779	1.17	1.38 (1.06-1.79)	1.28 (0.95-1.71)
3	11	13899	0.79	0.99 (0.54-1.79)	0.71 (0.33-1.49)
4+	6	4596	1.31	1.59 (0.71-3.56)	1.52 (0.63-3.68)
Severity of Poor Oral Health [e]					
None	304	406641	0.75	1 (reference)	1 (reference)
Mouth Ulcers, Toothache	41	53621	0.76	1.09 (0.78-1.50)	1.13 (0.79-1.61)
Bleeding/Painful gums, Loose teeth	84	106958	0.79	1.21 (0.95-1.54)	1.11 (0.84-1.46)
Dentures	283	162858	1.74	1.59 (1.35-1.88)	1.43 (1.18-1.73)
Competing Risk Model,[g] n=438,762					
Number of Existing Conditions					
None	1,470	2,756,088	0.53	1 (reference)	1 (reference)
1	1,603	1,365,174	1.17	1.45 (1.35-1.56)	1.32 (1.21-1.43)
2	370	318,934	1.16	1.57 (1.40-1.76)	1.41 (1.24-1.61)
3	90	74,911	1.20	1.70 (1.37-2.10)	1.56 (1.23-1.97)
4+	35	24,844	1.41	1.93 (1.38-2.70)	1.69 (1.15-2.48)
Severity of Poor Oral Health [e]					
None	1,470	2,756,088	0.53	1 (reference)	1 (reference)
Mouth Ulcers, Toothache	180	361,327	0.50	1.11 (0.95-1.29)	1.05 (0.88-1.25)
Bleeding/Painful gums, Loose teeth	426	705,237	0.60	1.22 (1.09-1.36)	1.14 (1.01-1.29)
Dentures	1,492	717,299	2.08	1.69 (1.57-1.83)	1.51 (1.38-1.65)

a. Computed per 1,000 person-years
b. Adjusted for age at enrollment, sex, race, and smoking history combined with pack-years
c. Adjusted for age at enrollment, sex, race, smoking history combined with pack-years, educational attainment, household income, alcohol consumption, obesity status, and history of chronic obstructive pulmonary disease
d. Not estimated due to insufficient sample size (<5 events)
e. Patients with multiple conditions were categorized based on their most severe condition reported: none, mouth ulcers/toothache (less severe), bleeding gums/painful gums/loose teeth, and dentures (most severe)
f. Former smokers who had abstained from smoking for more than 10 years
g. Treating smoking-related deaths as a competing risk; smoking-related mortality encompasses cancers of the lung, lip, pharynx, oral cavity, pharynx, esophagus, stomach, pancreas, larynx, cervix uteri (in women), kidney and renal pelvis, bladder, liver, colon, and rectum, and acute myeloid leukemia, as well as coronary heart disease, rheumatic heart disease, pulmonary heart disease, other forms of heart diseases, cerebrovascular disease, atherosclerosis, aortic aneurysm, additional arterial diseases, pneumonia, influenza, tuberculosis, and chronic obstructive pulmonary disease including emphysema, bronchitis, and chronic airways obstruction

Table S9. Sensitivity Analyses Combining Tracheal Cancer with Lung Cancer

	Cases, *n*	Person-years	Incidence Rate [a]	Hazard Ratio (95% CI) [b]	Hazard Ratio (95% CI) [c]
Bronchus and Lung Cancer					
Any Oral Health Condition	2,000	1,783,247	1.12	1.49 (1.39-1.59)	1.34 (1.24-1.45)
Dentures	1,428	716,878	1.99	1.63 (1.52-1.75)	1.47 (1.36-1.60)
Loose teeth	337	193,109	1.75	1.45 (1.29-1.62)	1.37 (1.21-1.56)
Painful gums	148	137,007	1.08	1.30 (1.10-1.54)	1.24 (1.03-1.49)
Bleeding gums	292	619,546	0.47	0.96 (0.85-1.09)	0.98 (0.85-1.12)
Toothache	140	205,907	0.68	1.00 (0.84-1.18)	0.93 (0.76-1.13)
Mouth ulcers	295	461,750	0.64	1.08 (0.96-1.22)	1.02 (0.89-1.17)
Bronchus, Lung, and Tracheal Cancer					
Any Oral Health Condition	2,002	1,783,264	1.12	1.49 (1.39-1.59)	1.34 (1.24-1.45)
Dentures	1,430	716,895	2.00	1.63 (1.52-1.76)	1.48 (1.36-1.60)
Loose teeth	337	193,109	1.75	1.44 (1.29-1.62)	1.37 (1.21-1.56)
Painful gums	148	137,007	1.08	1.30 (1.10-1.53)	1.24 (1.03-1.49)
Bleeding gums	292	619,546	0.47	0.96 (0.85-1.09)	0.97 (0.85-1.12)
Toothache	140	205,907	0.68	1.00 (0.84-1.18)	0.93 (0.76-1.13)
Mouth ulcers	295	461,750	0.64	1.08 (0.96-1.22)	1.02 (0.89-1.17)

a. Computed per 1,000 person-years
b. HR from multivariable Cox proportional hazards model, adjusted for age at enrollment, sex, race, and smoking history combined with pack-years
c. HR from multivariable Cox proportional hazards model, adjusted for age at enrollment, sex, race, smoking history combined with pack-years, educational attainment, household income, alcohol consumption, obesity status, and history of chronic obstructive pulmonary disease
